# Delirium in ICU patients is associated with increased temperature variability

**DOI:** 10.1186/cc12337

**Published:** 2013-03-19

**Authors:** AW Van der Kooi, TH Kappen, RJ Raijmakers, IJ Zaal, AJ Slooter

**Affiliations:** 1 University Medical Center Utrecht, the Netherlands

## Introduction

Delirium is an acute disturbance of consciousness and cognition. It is a common disorder in the ICU and associated with impaired long-term outcome [1,2]. Despite its frequency and impact, delirium is poorly recognized by ICU physicians and nurses using delirium screening tools [3]. A completely new approach to detect delirium is to use monitoring of physiological alterations. Temperature variability, a measure for temperature regulation, could be an interesting parameter for monitoring of ICU delirium, but this has never been investigated before. The aim of this study was to investigate whether temperature variability is affected during ICU delirium.

## Methods

We included patients in whom days with delirium could be compared with days without delirium, based on the Confusion Assessment Method for the ICU and inspection of medical records. Patients with conditions affecting thermal regulation, including infectious diseases, and those receiving therapies affecting body temperature were excluded. Twenty-four ICU patients were included after screening 334 delirious ICU patients. Daily temperature variability was determined by computing the mean absolute second derivative of the temperature signal. Per patient, temperature variability during delirious days was compared with nondelirium days using a Wilcoxon signed-rank test. With a linear mixed model, differences between delirium and nondelirium days with regard to temperature variability were analysed adjusted for daily mean Richmond Agitation and Sedation Scale scores, daily maximum Sequential Organ Failure Assessment score, and within-patient correlation.

## Results

Temperature variability was increased during delirium days compared with days without delirium (mean difference = -0.007, 95% CI = -0.004; -0.011, *P <*0.001). Adjusting for confounders did not alter our findings (adjusted mean difference = -0.005, 95% CI = -0.008; -0.002, *P <*0.001).

## Conclusion

Temperature variability is increased during delirium in ICU patients, which reflects the encephalopathy that underlies delirium. Opportunities for delirium monitoring using temperature variability should be further explored. Particularly, in combination with electroencephalography it could provide the input for an objective tool to monitor delirium.
